# A preliminary study on applying holistic health care model on medical education behavioral intention: a theoretical perspective of planned behavior

**DOI:** 10.1186/s12909-021-02746-0

**Published:** 2021-05-28

**Authors:** Liang-Miin Tsai, Yu-Hua Yan

**Affiliations:** 1 Superintendent Office, Tainan Municipal Hospital (Managed by Show Chwan Medical Care Corporation), No. 670, Chung Te Road, Tainan City, 701 Taiwan; 2 Department of Hospital and Health Care Administration, Chia Nan University of Pharmacy and Science, No.60, Sec. 1, Erren Rd., Rende Dist, Tainan City, 71710 Taiwan

**Keywords:** Holistic health care, Medical education, Behavioral intention, Postgraduate general clinic medicine training

## Abstract

**Background:**

This study aimed to apply the theory of planned behavior to identify the medical education behavioral intention of holistic healthcare on teachers and students who influence the medical and healthcare profession, as a revised future direction for hospitals to continue to implement holistic healthcare.

**Methods:**

This cross-sectional study was performed from April to May 2020. A questionnaire survey was conducted with the clinical teachers and students of the medical and healthcare profession in an individual Taiwan hospital as study subjects, based on judgmental sampling, and the study tool was a structured questionnaire.

**Results:**

A total of 360 valid samples were collected, 105 clinical teachers (29.2%) and 255 students (70.8%). Demographic variables were significantly different between clinical teachers (mean = 3.91) and students (mean = 3.73) (*p* = 0.023). Post hoc analysis regarding work experience found that those with work experience < 2 years (mean = 3.94) had higher results than those with 6–10 years (mean = 3.61) (*p* = 0.019). The results of multiple regression analysis indicate that the factors affecting medical education behavioral intention are subjective norm (t = 3.571, *p* < 0.001) and perceived behavioral control (t = 11.870, *p* < 0.001).

**Conclusions:**

With respect to medical education behavioral intention, clinical teachers and students are affected by the subjective norm and perceived behavioral control. It is recommended that, in the curriculum of holistic medical education, designing holistic medical education teaching templates and check forms can encourage clinical teachers to re-examine their beliefs in teaching, learning, and knowledge. The results of this study allow the advocator to consider from a broader view making policies of and promoting the platform of holistic healthcare on medical education. It is recommended that future researchers conduct research, investigation, and analysis on other stakeholders.

## Background

Holistic healthcare is a medical care model that was proposed by the Ministry of Health and Welfare (Taiwan) in 2005 when it was promoting the “Holistic Health Care Plan.” Not only does it emphasize the need to provide correct and effective prevention methods and patient-centered medical care, it also stresses the need for correct and dignified rehabilitation and support during illness [1]. With the evolution of human civilization, people have been constantly seeking progress, as has medicine. Therefore, the concept of holistic medicine has been gradually gaining attention in the medical field [2, 3]. Recently, after many reflections and reviews, medical circles at home and abroad have returned to a holistic view, emphasizing that complete medical care must cover a person’s physical, mental, and spiritual needs and that we must attach importance to his/her social adaptation. However, to implement holistic medical education, attention must be paid to every stage, from basic education of medical staff to continued education after graduation, from acute medical care to chronic long-term care, and from medical centers to community primary medical care [4, 5].

In addition to understanding the patient’s physical pain during the training process of a medical care team, psychological pressure, family burden, and re-adaptation of social roles all require education and training [6]. Providing holistic healthcare by a cross-disciplinary team can help resolve patients’ fears of disease and treatment [7] and deliver more complete holistic healthcare. Concurrently, it also allows professionals to continue to grow at work and improve the quality of medical care [8]. The ultimate goal is to promote patient’s recovery and return to the normal quality of life [9, 10].

The theory of planned behavior (TPB) is the most extensive and influential theory in human behavioral research [11–14]. The theory is derived from the theory of reasoned action proposed by Fishbein and Ajzen [15]. Ajzen [16] amended the missing parts of the theory of reasoned action and increased its explanatory power. TPB comprises factors of “attitude (AT),” “subjective norm (SN),” and “perceived behavior control (PBC)” and in the end “behavioral intention (BI)” [16, 17]. Moreover, “attitude” means that an individual has a positive or negative evaluation of a specific behavior. “Subjective norms” refer to the individual feeling of social pressure from other individuals or groups. “Perceived behavior control” refers to the degree of simplicity or difficulty that an individual perceives when performing a specific behavior. “Behavioral intention” is the result guided by the decision process of behavior choice [18].

The TPB has been continuously verified by researchers in different fields over the years and widely used in various research topics [19]. In the field of medical care, these topics include disease treatment behavior (insulin injection intention) [20]; intention of physicians and non-physicians to protect the privacy of electronic medical records [21]; behavioral intention of licensed pharmacists in hospitals to avoid erroneous dispensing [22]; attitude, subjective norm, and PBC influencing his/her intention for oral hygiene care [23]; and a hospital manager’s attitude and PBC influencing the intention of empirical management [24].

Previously, many researches on holistic medical education and training at home and abroad were mostly focused on research on medical knowledge and teaching techniques [25–27]. There is no empirical research on how the promotion of holistic medical education is affected by those factors affecting their behavioral intentions. This study still awaits further in-depth research by scholars. The main problem with holistic medical education and training is that each hospital still has its own exploration and lacks complete effectiveness evaluation based on the view that holistic medical education and training plays a greatly important role in the development of physicians or medical staff and that it can provide patients with more complete holistic medical care.

This study primarily aimed to apply the TPB to identify the factors influencing the behavioral intention of medical and healthcare clinical teachers and students toward promoting holistic healthcare in hospitals. This strategy is not featured in published medical educational literature, although promising research is emerging from other disciplines. Under the concept of general medicine, we can complete the learning goals of medical education and training of holistic healthcare and combine the TPB, as a revised future direction for hospitals to continue to implement holistic healthcare. Concurrently, it can also make up for the gaps in previous studies.

## Methods

### Design, participants, and procedures

The survey period of this study was from April 1, 2020 to May 31, 2020. The subjects of the study were clinical teachers and students in the medical and healthcare profession from a medical care corporation hospital in Southern Taiwan. This cross-sectional study was conducted using judgmental sampling, and the study tool was a structured questionnaire. The clinical teachers and students had a free will to decide whether they were willing to participate in this study. According to the hospital statistics, there were approximately 419 clinical teachers and students in the medical and healthcare profession, and the questionnaires were distributed to them.

Distribution and collection of questionnaires were conducted by the researchers. Teachers and students who agreed to participate in the medical and healthcare professions of this study were included in the survey, and those who do not meet the acceptance criteria of this study were excluded. All participants were informed about the study’s aim, and their consent for participation was obtained at the beginning of the study. The participants filled in the basic information and various questionnaire assessments, and the researcher tracked the participant’s questionnaire fill-in situation and checked whether the interviewee’s record was complete when receiving the information to reduce the missing value of the data.

### Sample size

According to Dillman [28], the sample size can be estimated using the following formula: ${N}_{SAMPLE}=\frac{\left({N}_P\right)(p)\left(1-p\right)}{\left({N}_P-1\right){\left(E/C\right)}^2+(p)\left(1-p.\right)}$

*N*_*SAMPLE*_ is the sample size; *N*_*P*_ is the population size; *E* is the tolerance error; C is the Z value (1.96) of 95% confidence interval; and *p*, 1 − *p* is the population variance. Under consideration of normal distribution and fitting expected characteristic, the maximum population variance was 50%. According to the formula above, the minimum standard of sample size was 201. The sample size was 360. The result (360) was more than the minimum standard (201). Hence, the number of sample was acceptable in this study.

### Study tools

The study tool was a structured questionnaire. We formulated the questionnaire scale with references to relevant literature, current operations, and expert interviews on designing questions, using the scoring method of the five-point Likert scale. The participants were instructed to respond on a five-point Likert scale in numerical values: 5 (strongly agree), 4 (agree), 3 (neutral), 2 (disagree), and 1 (strongly disagree), for all statements. Questionnaires used in this study included the following: (1) eight questions on basic demographic and sample characteristics data (sex, age, education level, marital status, practice discipline or department, clinical teacher qualification, current job title, and current years of services) and (2) 26 questions on participation behavior and medical education behavioral intention (attitude, subjective norm, PBC, and medical education behavioral intention), with a total of 34 questions.

Regarding the compilation of measurement question group based on the TPB construct, first, the measurement method recommended by the theory was used as basis before relevant domestic and foreign literature was consulted. Relevant question groups were drafted on the researcher’s own medical work experience. Relevant opinions and questions were collected through interviews with clinical teachers and students. Following the compilation of the first draft of the questionnaire, the opinions of three experts were sought. According to the recommendations of these experts, questions were added, deleted, and revised to strengthen the content validity.

### Validity and reliability analysis

We used Cronbach’s α analysis to analyze the internal consistency of the measurement variables of each dimension to understand the reliability of the measurement dimension. Generally speaking, Cronbach’s α value > 0.9 represented a high reliability value. The analysis showed the following results: Cronbach’s α values in “attitude,” 0.957; “subjective norm,” 0.950; “perceived behavior control,” 0.949; and “medical education behavioral intention,” 0.946. Cronbach’s α value for each scale and the overall questionnaire was 0.973, all of which were > 0.9, indicating that the questionnaire in this study had very good reliability.

Experts were invited to conduct a validity analysis, and the expert consensus calculated the content validity index (CVI) as 0.98, which is extremely efficient. The CVI value of each item on the four scales ranged from 0.80 to 1, indicating that the questionnaire in this study had very good validity.

### Ethics approval and consent to participate

This study adheres to the guidelines of the Declaration of Helsinki. This study was granted an ethics waiver by the Institutional Review Board at Show Chwan Memorial Hospital (IRB1090203). According to the same committee, formal written informed consent was not required.

Similarly, the study was deemed as “not involving human subjects research” by the Institutional Review Board at Show Chwan Memorial Hospital Human Subjects Committee and exempted from human subjects’ oversight. The first author invited all participants by email or telephone, emphasizing that participation was voluntary and anonymous. All participants provided oral consent.

### Statistical analysis

After the valid questionnaire data were decoded and input, statistical analysis was conducted with the SPSS 22.0 statistical package as the tool. All data were checked and screened with descriptive statistics, and errors were eliminated before statistical analysis was performed.

## Results

In this study, 419 questionnaires were sent to the clinical teachers and students in the medical and healthcare profession, and a total of 364 questionnaires were retrieved. After four invalid questionnaires were eliminated, 360 valid questionnaires were obtained, with a recovery rate of 85.9%. Relevant basic data analysis is shown in Table 1. Among the 360 questionnaires retrieved, 257 were women (71.4%). There were 105 clinical teachers (29.2%) and 255 students (70.8%). Most of them were aged 41–50 years (34.2%), had university degrees (86.1%), and were unmarried (50.6%). For those with working experience of > 10 years (46.7%), 100 were in the medical departments (27.8%) and 260 were in the healthcare departments (72.2%).

**Table 1:** Characteristics of the study population (*n* = 360)

Item	N	%
Sex		
Female	257	71.4
Male	103	28.6
PGY		
Clinical teachers	105	29.2
Trainee	255	70.8
Job title		
Manager	100	27.8
Staff	260	72.2
Age (years) Mean: 38.9		
< 30	96	26.9
31–40	94	26.1
41–50	123	34.2
> 50	47	13.1
Education level		
Bachelor’s degree	310	86.1
Master’s degree	50	13.9
Marital status		
Single	182	50.6
Married	178	49.4
Job tenure (year)		
≤ 2	102	28.3
3–5	48	13.3
6–10	42	11.7
> 10	168	46.7
Category		
Medical personnel	260	72.2
Physicians	100	27.8

The scale scores of the four dimensions of this study were as follows: Attitude average was 4.21 ± 0.62 points, subjective norm average was 4.04 ± 0.68 points, perceived behavioral control average was 3.65 ± 0.73 points, and behavior intention average was 3.78 ± 0.66 points. In terms of attitude, the scale of “holistic medical education is valuable” scored the highest (4.3 points), and the scale of subjective norm was “the hospital will arrange teaching holistic medical education courses,” with 4.09 the highest. Perceived behavioral control had the highest score of 3.77 for “I encounter problems in teaching (or learning) holistic medical education courses, and it is easy to get support from others.” Behavior intention had the highest score of 3.9 for “I am motivated to study holistic medical education courses”.

To understand the relationship between the study variables, this study used the Pearson correlation matrix to determine the degree of correlation between various dimensions. The results of the study are shown in Table 2. The correlation coefficient r was 0.843 between attitude and subjective norm, 0.599 between attitude and PBC, and 0.591 between attitude and medical education behavioral intention. The degree of correlation between the two groups reached a positive correlation of high significance (*p* < 0.001).

**Table 2:** Correlation (Pearson correlation analysis)

Item	AT	SN	PBC	BI
1. Attitude	1			
2. Subjective norm	.843***	1		
3. Perceived behavioral control	.599***	.708***	1	
4. Behavior intention	.591***	.682***	.758***	1

***p < .001

### Differences in medical education behavioral intentions among different demographic backgrounds and sample characteristics

In this study, t-test and single factor variance analyses were used to determine the differences in medical education behavioral intentions among different demographic backgrounds and sample characteristics (Table 3). The results demonstrated that there was a significant difference between clinical teachers and students (*p* < 0.05) and the variable “years of working” also showed a statistically significant difference (*p* < 0.05). After Scheffé post hoc analysis was applied, it was found that those with a working experience of < 2 years had higher scores than those with a working experience of 6–10 years (*p* < 0.05).

**Table 3:** Differences in medical education behavioral intentions among different demographic backgrounds and sample characteristics (*n* = 360)

Item	N	Mean	SD	*P*-value	Scheffé
Sex				.999	
Female	257	3.78	.61		
Male	103	3.78	.76		
PGY				.023**	
Clinical teachers	105	3.91	.67		
Trainee	255	3.73	.65		
Job title				.890	
Manager	100	3.78	.65		
Staff	260	3.79	.68		
Age (years) Mean: 38.9				.051	
< 30	96	3.91	.59		
31–40	94	3.69	.69		
41–50	123	3.72	.62		
> 50	47	3.87	.75		
Education level				.362	
Bachelor’s degree	310	3.77	.65		
Master’s degree	50	3.86	.69		
Marital status				.187	
Single	182	3.83	.62		
Married	178	3.74	.70		
Job tenure (year)				.019**	(1) > (3)
≤ 2 (1)	102	3.94	.68		
3–5 (2)	48	3.70	.58		
6–10 (3)	42	3.61	.70		
> 10 (4)	168	3.75	.64		
Category				.969	
Medical personnel	260	3.78	.77		
Physicians	100	3.78	.61		

** *p* < .01

### Distribution of attitude, subjective norm, PBC, and medical education behavioral intention

Once the correlation among the various dimensions was understood through correlation analysis, this study used regression analysis to explore the degree of influence of attitude, subjective norm, and PBC on medical education behavioral intention. The results of the multiple regression analysis shown in Table 4 demonstrated that the overall F-test value was 191.24 (*p* < 0.001), indicating the significance of using attitude, subjective norm, and PBC as predictive variables to predict the overall and medical education behavioral intentions. These variables explain the variation (61.40%) in the overall medical education behavioral intention and significance level. Among the influences of various predictive variables on the overall medical education behavioral intention, PBC (β = 0.552, *p* < 0.001) had the greatest influence, followed by subjective norm (β = 0.247, *p* < 0.001), whereas attitude (β = 0.052, *p* = 0.392) did not reach a significance level. Moreover, the variance inflation factor of each predictive variable was < 10, which indicated that there was no multicollinearity problem among the three predictive variables.

**Table 4:** Prediction and explanatory power of each predictive variable on the overall behavioral intention

Item	Standardized coefficient β	t value	*P*-value	Collinearity diagnostics	
				Tolerance	VIF
Constant		5.251	<.001		
Attitude	.052	.857	.392	.290	3.448
Subjective norm	.247	3.574	<.001	.225	4.439
Perceived behavioral control	.552	11.870	<.001	.498	2.007
*R*^2^ = 0.617	Adj *R*^2^ = 0.614	F = 191.24	*P* < 0.001		

## Discussion

To implement holistic healthcare, it is necessary to adopt a sound medical education system, appropriate learning fields, and dedication of clinical teachers to holistic medical education. This study is based on the TPB by Ajzen [17], which, in a broad sense, is supported by experience. Through behavioral attitude, subjective norm, and PBC, the intentions of various types of behaviors can be predicted. Similar to previous studies, this study shows that, in physicians and medical staff, there is high correlation between the influences of “subjective norm” and “perceived behavior control” on medical education behavioral intention. Thus, if a hospital has a system that requires clinical teachers to guide students to understand the needs (physical, psychological, spiritual, and social) of patients, their medical education behavioral intention could be higher. However, this result has not been previously described. The existence of institutional norms is closely related to the development of holistic medicine, and how to promote the willingness of “people” to implement “systems” will be a challenge.

Moreover, the more a teacher thinks that he/she is in possession of professional knowledge, teaching resources, and environmental opportunities, the more likely that he/she is self-confident, which affects perceived behavior, leading to heightened intention to implement holistic medical education. The study of Pong [29] also confirmed critical factors for the successful implementation of holistic medical education through experiential learning. Therefore, in the future, if we were to increase the behavioral intention of a teacher to implement holistic medical education, we should start by controlling this aspect of perceived behavior.

Furthermore, in the past, researchers believed that “behavioral intention” was determined by three factors, namely, “attitude,” “subjective norm,” and “perceived behavior control” [16, 17]; however, according to the results of this study, “attitude” does not have a significant influence on “behavioral intention,” the possible reason being that clinical teachers have yet to undergo positive or negative evaluations on the implementation of holistic medical education. This outcome is contrary to that of Guo et al. [24], who found that education positively moderated the relationship between attitudes and intention to use evidence-based management. Holistic medical care is a medical care model, whereby the patient’s medical care is delivered by cross-disciplinary professional medical service providers, and exclusive medical plans are tailored made according to the patient’s life pattern, mental state, and various real-life needs. Therefore, if clinical teachers and students are encouraged to make positive evaluations of holistic medical education through various teaching mechanisms, the hospital’s satisfactory level with overall promotion of holistic medical education will increase.

Finally, there is a significant difference between postgraduate general clinic medicine training (PGY) clinical teachers or Trainee and work experience in medical education behavioral intention. One possible reason for this is that the hospital has only recently introduced holistic medical education and training. Clinical teachers may be busy at work while responsible for teaching, and the workload of clinical teachers and students is different. As a result, students have different behavioral intentions for holistic medical education. Ottenhoff-de Jonge et al. [30] showed that the educational beliefs of medical educators influence their teaching practices. It is possible that, if the hospital can appropriately adjust and improve the workload of clinical teachers, it will help the implementation of holistic medical education and potentially help the development of clinical teachers’ work.

### Implications for teaching and future research

Current research results show that subjective norms and perceptual behavior control have an impact on medical education behavior. Therefore, in the curriculum of holistic medical education, designing holistic medical education teaching templates and check forms can help them realize their beliefs and can encourage clinical teachers to re-examine their beliefs in teaching, learning, and knowledge. Reflecting on how to determine which holistic medical knowledge is relevant to learning, creating a positive learning atmosphere, and cultivating students’ internal motivation are critical to the quality of our education. The healthcare professionals of the future are of major importance for the quality of our education of future healthcare professionals.

Further research is needed to investigate the extent to which the beliefs of the hospital manager can change and develop toward holistic healthcare model-centeredness. For such a study, the presented framework can provide a useful instrument.

### Additional limitations

This study only focuses on the clinical teachers and students in the individual hospital as study subjects, and the conclusion may not be inferred to other hospitals; hence, there is a rather limited extrapolability. It is suggested that, in future studies, samples should be expanded to hospitals of various levels to improve the extrapolability of the study results. Moreover, the reasons for the variables (e.g., attitude) that have not received empirical support in this study still require further qualitative studies, such as field interviews and secondary data collection. Finally, as the subjects of this study are clinical teachers and students, it is recommended that future researchers conduct research, investigation, and analysis on other stakeholders (e.g., hospital operators).

## Conclusions

Based on the results of this study, it is recommended that hospitals apply the following methods when implementing holistic medical education: 1. cross-disciplinary discussions and meetings in relation to holistic medical education, in which students (trainees) may practice and participate in interdisciplinary holistic healthcare; 2. cross-disciplinary team learning to create a cooperative relationship of providing care together; 3. learning platform that provides clinical teachers and students with more holistic medical education and training; and 4. the establishment of system standards can make the implementation of holistic medical education more practical.

## Data Availability

Data cannot be made publically available owing to the fact that the privacy of individual participants cannot be compromised. However, the dataset is available from the corresponding author on reasonable request.

## Abbreviations

PGY: Postgraduate general clinic medicine training
CVI: Content validity index
TPB: The theory of planned behavior
AT: Attitude
SN: Subjective norm
PBC: Perceived behavior control
BI: Behavioral intention
